# The Royal British College of Nursing

**Published:** 1917-02-17

**Authors:** 


### THE MATRONS' AND SISTERS' DEPARTMENT.

### THE ROYAL BRITISH COLLEGE OF NURSING.

### The Central Committee's Policy of Disunion Demolished.

It is remarkable, but not surprising, to find that at the moment when the whole nation and Empire are united to overthrow and defeat a policy of evil, injustice, and tyranny, when political party strife is at a discount, the representatives of working nurses, being members of the Manchester Branch of the National Union of Trained Nurses, should have united to censure its Executive Committee for introducing politics and sowing disunion, by severing its negotiations with the College of Nursing, without reference to the opinion of the branches and their members. In a meeting of eighty-six nurses, eighty-one supported with acclamation the condemnation of disunion. In the fate of such an overwhelming vote by independent working nurses of the highest intelligence, following the repudiation by the Royal British Nurses' Association and by the greatest Scottish organisation of nurses, the Association for the Promotion of the Registration of Nurses in Scotland, of the policy of severance and "divide" pursued by the Central Committee for the State Registration of Nurses, the hollowness of the claim of the Central Committee to be a representative body (?) and so superior to the Council of the Royal British College of Nursing becomes manifest. The National Union of Trained Nurses is one of the so-called representative bodies, which the Central Committee insisted should be included as a "constituent society" under its Bill, to elect the members of the Provisional Council. When the College refused to agree to this proposal because of its unconstitutional character, the hollowness of which the Manchester vote exposes, the Central Committee broke away from the College and declined to co-operate further in working for an agreed Bill.

At the Manchester meeting the chairwoman of the Executive Committee of the National Union, which was censured by its nurse-members on the 10th instant, in the course of her speech, asked the nurses, with engaging frankness, "Do you think that if the Central Committee had nominated me as an individual, being the matron of a small cottage hospital in Surrey, that I should have carried the slightest weight?" The accuracy of this amusing forecast of the real feeling of the working nurses, on this point, was demonstrated by the overwhelming vote recorded later by the nurses present. The chairwoman of the Executive Committee of the Union proceeded to rub this point in by declaring that "Had I been nominated by the Central Committee as the representative of the National Union of Trained Nurses, should not I have carried weight?" She added, "Now this is just the point of difference in the two Bills." The chairwoman of the Union Executive is entirely right, and we owe her grateful thanks for having brought out the real underlying reasons why the Central Committee will not agree to give the names of the representatives of the so-called constituent societies. Demonstrably, the chairwoman of the Union Executive is precisely accurate in declaring in her statement, that, the printing of the names in the Bill would be fatal to their holders' chance of election by the whole body of nurses. The accuracy of this was demonstrated at the Manchester meeting.

In the face of the truth, as confessed by the chairwoman of the National Union Executive, and the true inwardness of the meaning of the policy of division represented by the Central Committee for the State Registration of Nurses she reveals, is there a single independent member on the Central Committee, who can face the nursing profession and justify the further continuance of a "Kill the Bill" policy? Yet that policy constitutes at the moment, practically, the only reason why Parliamentary sanction should not be obtained for an agreed bill, creating a State Register and the Statutory Recognition of the British nursing profession. There is therefore no justification for the continuance of the "Kill the Bill" policy of the Central Committee.

